# Assessment of veterinary drugs in plants using pharmacokinetic approaches: The absorption, distribution and elimination of tetracycline and sulfamethoxazole in ephemeral vegetables

**DOI:** 10.1371/journal.pone.0183087

**Published:** 2017-08-10

**Authors:** Hui-Ru Chen, Tirawat Rairat, Shih-Hurng Loh, Yu-Chieh Wu, Thomas W. Vickroy, Chi-Chung Chou

**Affiliations:** 1 Department of Veterinary Medicine, College of Veterinary Medicine, National Chung Hsing University, Taichung, Taiwan; 2 Department and Graduate Institute of Pharmacology, National Defense Medical Center, Taipei, Taiwan; 3 Department of Physiological Sciences, College of Veterinary Medicine, University of Florida, Gainesville, Florida, United States of America; Huazhong Agriculture University, CHINA

## Abstract

The present study was carried out to demonstrate novel use of pharmacokinetic approaches to characterize drug behaviors/movements in the vegetables with implications to food safety. The absorption, distribution, metabolism and most importantly, the elimination of tetracycline (TC) and sulfamethoxazole (SMX) in edible plants *Brassica rapa chinensis* and *Ipomoea aquatica* grown hydroponically were demonstrated and studied using non-compartmental pharmacokinetic analysis. The results revealed drug-dependent and vegetable-dependent pharmacokinetic differences and indicated that ephemeral vegetables could have high capacity accumulating antibiotics (up to 160 μg g^-1^ for TC and 38 μg g^-1^ for SMX) within hours. TC concentration in the root (C_max_) could reach 11 times higher than that in the cultivation fluid and 3–28 times higher than the petioles/stems. Based on the volume of distribution (Vss), SMX was 3–6 times more extensively distributed than TC. Both antibiotics showed evident, albeit slow elimination phase with elimination half-lives ranging from 22 to 88 hours. For the first time drug elimination through the roots of a plant was demonstrated, and by viewing the root as a central compartment and continuous infusion without a loading dose as drug administration mode, it is possible to pharmacokinetically monitor the movement of antibiotics and their fate in the vegetables with more detailed information not previously available. Phyto-pharmacokinetic could be a new area worth developing new models for the assessment of veterinary drugs in edible plants.

## Introduction

Antibiotic drugs have long been used for the treatment or prophylaxis of bacterial infections in humans and non-human species. Although there is substantial variation in the manner in which antibiotics are eliminated by different animal species, it is estimated that a significant fraction (30–90%) of the total dose of administered antibiotics are excreted in feces or urine as either the parent drug or metabolites [1]. While the majority of drugs excreted from humans pass through wastewater treatment facilities prior to environmental release, drug removal or destruction during usual treatment processes is incomplete, which gives rise to detectable quantities of antibiotics in sewage effluents [2–5]. In contrast to human drugs, veterinary antibiotics that are used in livestock industries can contribute more significantly to environmental drug residues when manure from treated animals is used for soil conditioning or fertilizer. Furthermore, there is significant potential for veterinary antibiotics to be transported from land to surface water as a result of runoff from rainfall or direct seepage into ground water supplies from contaminated soils [2,6–7]. One of the major concerns regarding the presence of antibiotics in terrestrial and aquatic environments is the heightened potential for increased resistance to antibiotics among pathogenic microorganisms [8–9], as well as detrimental effects on ecosystems and human health. In addition, since some drugs are chemically stable and can persist within the environment for relatively long periods of time, there is an added risk of drug accumulation by plants that are grown on heavily contaminated soils or watered with contaminated water sources [1,6,10]. Such accumulation in edible plants poses an unknown health risk to individuals who consume such plants.

Tetracyclines (TCs) and sulfonamides (SAs), which are among the most extensively used veterinary antibiotics [1,10,11], are highly excreted with as much as 40–90% of orally administered doses eliminated in urine or feces [8,10]. In light of this, it is not surprising that TCs and SAs are frequently detected in the manure, soil, and water in the vicinity of intensive commercial livestock operations. Drug concentrations in manure have been reported as high as 81 μg kg^-1^ and 101 μg kg^-1^ for tetracycline (TC) and sulfamethoxazole (SMX), respectively [7], with comparable or even higher soil concentrations reported for both TC (74–300 μg kg^-1^) and SMX (0.9–55 μg kg^-1^) [10,12,13].

For most drugs belonging to the TCs class, soil adsorption coefficients (K_d_) are quite high (approx. 400–3000 L kg^-1^) and are several orders of magnitude greater than the SA-class agents (approx. 0.6–5 L kg^-1^) [6,10,14]. In light of the strong adsorption of TCs to most soils, the transport efficiency from soil into water is much lower for TCs compared to SAs. These differences may explain in part why higher soil levels of TCs (compared to SAs) do not yield corresponding higher drug levels in aquatic environments. For example, similar concentrations of TC (0.11–1.57 μg L^-1^) [15–17] and SMX (0.48–1.9 μg L^-1^) have been reported in surface water samples in spite of much higher TC levels in accompanying soils [10,18–20]. In Taiwan, several studies have uncovered high concentrations of SMX in soil samples (5.82–7.35 μg L^-1^) [15,16] and ground water (0.007–0.47 μg L^-1^) in the vicinity of animal husbandries [13,19], whereas levels of TC were either very low (0.005 μg L^-1^) [13] or undetectable in surface water [19,21].

The impact of these drugs on the plants appear to be minor with minimal detrimental effects on plant growth and photosynthesis rates. For example, wheat and alfalfa survive exposure to oxytetracycline (OTC) at concentrations of 37 mg L^-1^ [22] and 90 mg L^-1^ [23], respectively. Barley endures exposure to sulfamethazine (SMT) and sulfadimethoxine (SDM) at 11.5 mg L^-1^ [24] while lettuce, tomatoes, carrots, and cucumber plants can endure SMT or TC at concentrations up to 300 mg L^-1^ [25]. Therefore, plant can survive and grow in antibiotic-contaminated environment.

Because of the potential to contaminate plants that enter the human food chain, investigators have studied the accumulation of TCs and SAs in a variety of edible plants grown in antibiotic-contaminated soils or solid media [13,26–33] or water [34–37]. However, to the best of our knowledge, other than accumulation no studies have examined the fate of drug in edible plant in a pharmacokinetic way, including rates and extent of drug distribution or possible presence of elimination processes by plants, including drug metabolism. Therefore, the aim of this study was to establish a model to investigate and characterize pharmacokinetic processes (absorption, distribution, metabolism, and elimination) of TC and SMX in edible plants, namely Chinese cabbages (*Brassica rapa chinensis*) and water spinach (*Ipomoea aquatica*). These plants were chosen as phyto-pharmacokinetic model because it grows rapidly and is considered one of the most popular vegetables, especially in Eastern and Southeastern Asia. In addition, preliminary field investigations were undertaken to examine the presence and levels of TC and SMX residues in *B*. *rapa chinensis* and *I*. *aquatica*.

## Materials and methods

### Chemicals and reagents

All of the chemicals used in this study were reagent grade or higher. Tetracycline hydrochloride (TC) and sulfamethoxazole (SMX) were purchased from Sigma Co. (St. Louis, USA). Oxalic acid dihydrate (C_2_H_2_O_4_·2H_2_O) and disodium hydrogen phosphate anhydrous (Na_2_HPO_4_) were from Panreac Chemical Co. (Saxony-Anhalt, Germany); EDTA·2Na (C_10_H_14_N_2_O_8_Na2·2H_2_O) was from Fluka Chemika (Buchs, Switzerland). Solutions were freshly prepared in ultra-pure water (resistivity >17 MΩ cm).

### Experiment materials

Chinese cabbage (*B*. *rapa chinensis*) and water spinach (*I*. *aquatica*) seedlings were obtained from local greenhouse in Beitun District of Taichung City, Taiwan. The vegetable seedlings were grown on sponge cubes (2.5 x 2.5 x 2.5 cm) in cups in a climate-controlled greenhouse for a period of 4 weeks, at which time 5 separate leaves of *B*. *rapa chinensis* had emerged and the average plant weight of *B*. *rapa chinensis* and *I*. *aquatica* were 27.3 g and 13.0 g, respectively. Plants were then transferred to the laboratory where the roots were rinsed with tap water prior to being transferred into cultivation fluid (see below) for growth and pharmacokinetics studies.

### Hydroponic experiment setting

The cultivation fluid used for this study was slightly modified from Kimura B solution [38]. The composition of the basic nutrient solution was (all concentrations expressed in mg L^-1^): 24.1 (NH_4_)_2_SO_4_, 9.25 KNO_3_, 67.5 MgSO_4_·7H_2_O, 12.4 KH_2_PO_4_, 7.5 C_6_H_5_FeO_7_, 43.1 Ca(NO_3_)_2_·4H_2_O, 0.5 mL 1 N HCl, 1.55 H_3_BO_3_, 0.34 MgSO_4_·4H_2_O, 0.58 ZnSO_4_·7H_2_O, 0.13 CuSO_4_·5H_2_O, and 0.08 H_2_MoO_4_. The pH was adjusted to 7.0 with NaOH. The experimental plants were placed into 500-mL plastic beakers that were covered with aluminum foil in order to prevent roots from any direct light exposure. Each vessel contained 100 mL of cultivation fluid and the plants were allowed to acclimate to these conditions for 5 days prior to starting the trials. All plants were cultivated under natural photoperiod which was approximately 12 hr-light/12 hr-dark.

### Phyto-phamarcokinetic studies

For drug absorption and distribution studies, 25 plants (for each species) were grown in cultivation solution containing TC or SMX 100 μg mL^-1^ (n = 5 for each time point). The roots, leaves, and petioles/stems were collected at 0.5, 3, 6, 12 and 24 hr and analyzed for drug content by HPLC-UV (see below). Small aliquots (2 mL) of cultivation fluid were removed at each time point and stored at -70°C for subsequent drug analysis. At each predetermined time point, plants were removed from hydroponic solution and washed extensively (approx. 2 L running water) in order to remove drug residues from the root surface. Plants were blotted dry on paper towels, weighed and plant tissues (root, petiole and leaf) were separated with a razor blade. Five grams of each tissue part was ground with a pestle in a stainless steel mortar containing 10 mL of double-distilled water (DDW) and subjected to a liquid-liquid extraction procedure (see below). The bioaccumulation factor (BAF), defined as concentration of drug in plant tissue / concentration of drug in the cultivation fluid, was calculated for each part of the vegetable. The metabolism study was performed on a separate setting at the 5^th^ day after cultivation in drug-containing fluid; the presence of any drug-related metabolite was detected by LC/MS. For evaluation of drug elimination, after growing the plants in 100 μg mL^-1^ of antibiotic-containing solution for 24 hrs, the root was cleaned with 3 L of running DDW before being transferred to a new vessel containing drug-free cultivation fluid. The concentrations of antibiotics in the new cultivation fluid were determined at 0.5, 3, 6, and 12 hr. In a separate experiment, the cultivation fluid of *B*. *rapa chinensis* was replenished every 30 min for 10 times to determine how much drug is eliminated under those condition.

### Drug analysis

#### Liquid-liquid extraction

The TC and SMX were extracted from plant tissues using an extraction procedure modified from Li et al. [18] and Arikan et al [39]. In brief, approximately 2 g of freshly-ground plant tissue was transferred into 4 mL of extraction buffer (0.5 M oxalic acid: 1 M NaCl: 95% alcohol, 25:25:50, v/v/v) and vortex mixed vigorously for 1 min. Extracts were centrifuged (2500 rpm for 10 min) and supernatants were collected, filtered through 0.45-μm membrane and stored at 4°C for analysis.

#### HPLC analysis

The HPLC system consisted of a Waters 510 pump (Waters CO, Massachusetts, USA), a Shimadzu SIL-10A auto-injector (with SCL-10A system controller, Shimadzu, Kyoto, Japan) and a UV-VIS detector (Hitachi L-4250, Hitachi Ltd., Tokyo, Japan). Chromatographic separation for SMX was performed using a XBridge^™^ C18 column (4.6 x 250 mm, 5 μm, Waters Technologies, Inc. Dublin, Ireland). For TC analysis, chromatographic separation was performed using an Inspire C18 column (4.6 x 250 mm, 5 μm, DIKMA Technologies, Inc. CA, USA). Samples (20 μL) were injected and run at a flow rate of 1 mL min^-1^ in mobile phase (MeOH: ACN: 0.05M oxalic acid = 15:15:70 (v/v/v)) and detected at 360 nm. For SMX analysis, plant extracts (20 μL) were injected into the chromatography system and run at a flow rate of 1 mL min^-1^ mobile phase (ACN: 0.05 M phosphoric acid = 25:75 (v/v)) passing through the UV detector set at 270 nm. The LOD (s/n ≥3) was 0.1 μg mL^-1^ for TC and 0.05 μg mL^-1^ for SMX. The data was analyzed by Clarity^™^ (DetaApex Ltd., Czech Republic).

#### LC/MS analysis

The LC/MS method which used to identify structural analogs of TC and SMX was modified from Koesukwiwat et al. [40]. Briefly, the mass spectra were obtained by a Thermo TSQ Quantum Ultra EMR triple quadrupole mass spectrometer (Thermo-Scientific, San Jose, CA, USA). The instrument was operated using electrospray ionization (ESI) sources in positive mode. The MS/MS conditions were set as follows: spray voltage, 4.0 kV; capillary temperature, 35°C; sheath gas pressure (N_2_), 30 units; auxiliary gas pressure (N_2_), 5 units; collision gas (Ar), 1.5 mTorr; scan time, 0.5 s.

### Pharmacokinetic analysis

Pharmacokinetic parameters of TC and SMX in the roots, including maximum drug concentration (C_max_), terminal rate constant (λ_z_), elimination half-life (t_½_), area under the concentration-time curve (AUC), area under the first moment of the concentration-time curve (AUMC), mean residence time (MRT), apparent total body clearance (CL), and volume of distribution at steady state (Vss) were determined using non-compartmental model analysis by WinNonlin^®^ software (version 4.0.1, Pharsight, Mountain View, CA, USA).

### Field investigation of antibiotics residues

Samples of *B*. *rapa chinensis* (n = 35) and *I*. *aquatica* (n = 37) were either purchased from local markets or collected from lands near pig farms around different regions of Taiwan for determination of TC and SMX residues. The 2 veterinary antibiotics were extracted and determined by HPLC procedures described above.

### Statistical analysis

Where applicable, statistical analysis was performed with one-way analysis of variance (ANOVA) followed by the least significant difference (LSD) test for multiple comparisons. All comparisons were considered statistically significant if p-value <0.05.

## Results

### Absorption and distribution of TC and SMX

**Fig 1:**
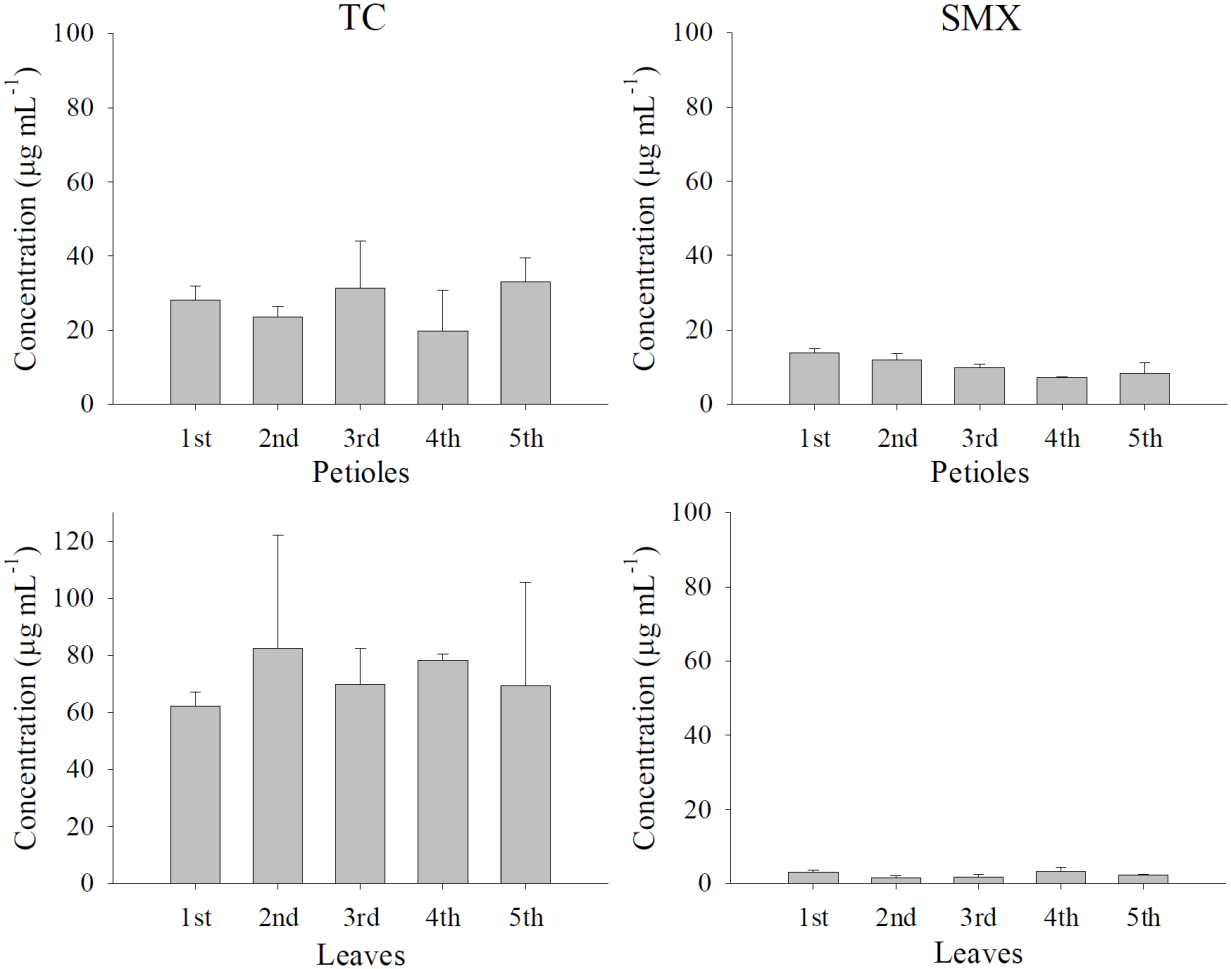
Comparison of TC (left panel) and SMX (right panel) accumulation among 5 petioles (top panel) and leaves (bottom panel) of *B*. *rapa chinensis* (n = 5) after 24 hrs of drug absorption. Noting that the 1^st^ petiole/leaf is the oldest while the 5^th^ is the youngest. Values represent means ± SEM.

Preliminary studies revealed that the absorption and distribution profiles of both antibiotics in *B*. *rapa chinensis* were similar at three concentrations (1, 10 and 100 μg mL^-1^) (data not shown), so the highest concentration (100 μg mL^-1^) was used for follow-up experiments. There were no statistical significant differences (p>0.05) in drug distribution among the 5 leaves/petioles of individual plants (Fig 1).

**Fig 2:**
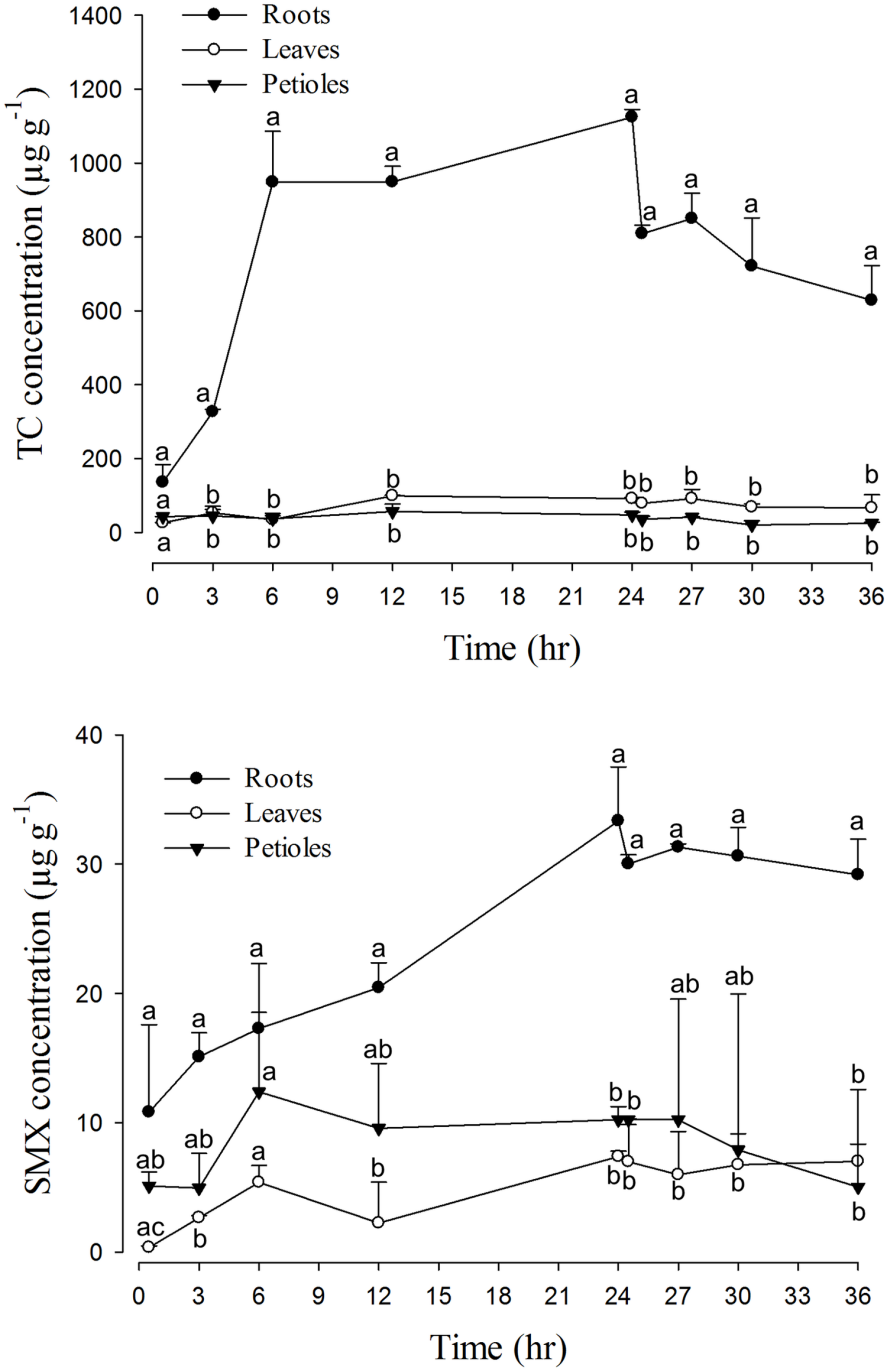
The combined concentration-time profiles of 24-hrs absorption and 12-hrs elimination of TC (upper panel) and SMX (lower panel) in different parts of *B*. *rapa chinensis* (n = 5). Values represent means ± SEM. Means in the same time point with different superscripts are significantly different from each other (p<0.05).

**Fig 3:**
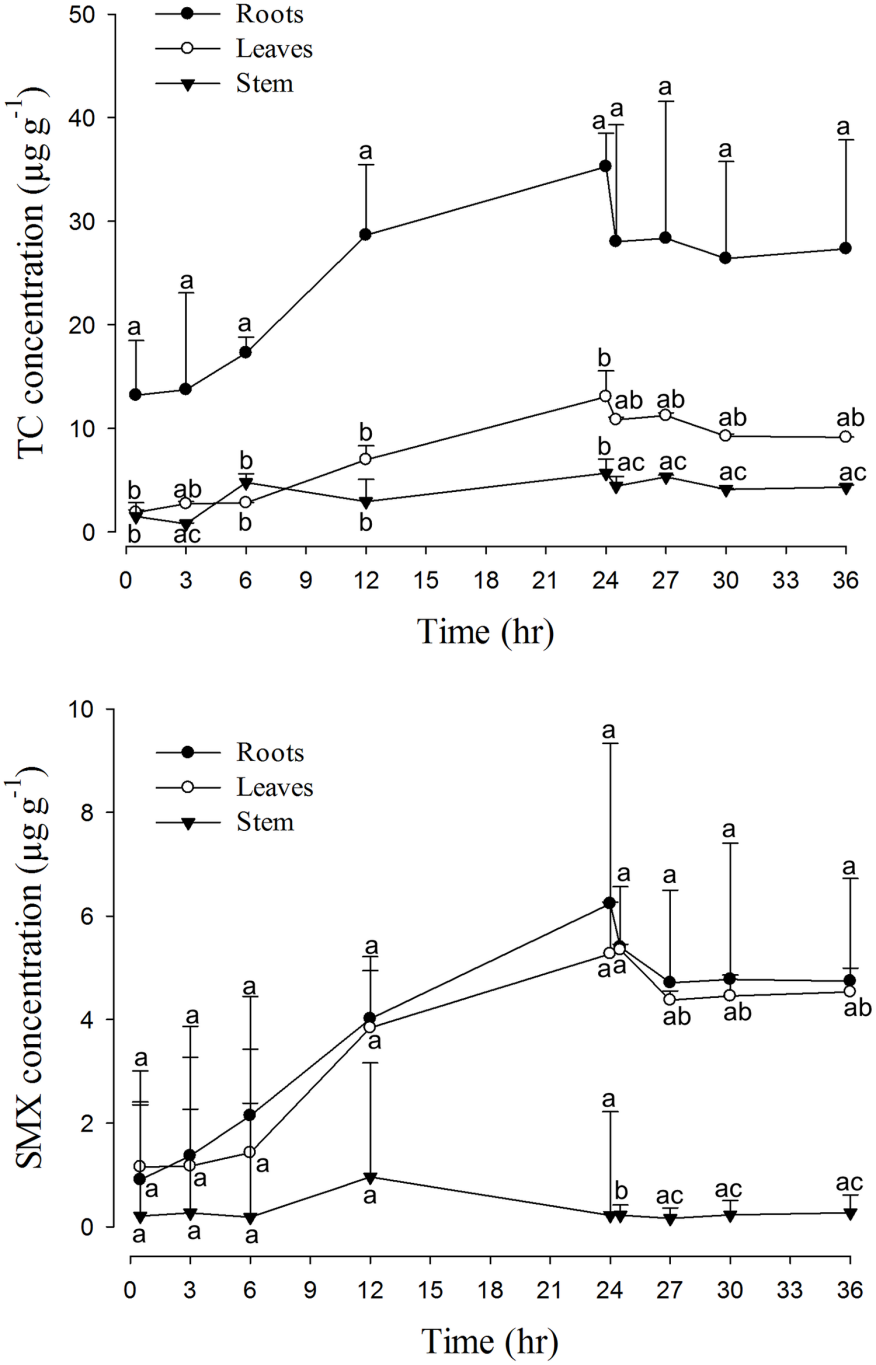
The combined concentration-time profiles of 24-hrs absorption and 12-hrs elimination of TC (upper panel) and SMX (lower panel) in different parts of *I*. *aquatica* (n = 5). Values represent means ± SEM. Means in the same time point with different superscripts are significantly different from each other (p<0.05).

The concentrations of TC or SMX in *B*. *rapa chinensis* and *I*. *aquatica* tissues reached significantly different levels and exhibited different distribution patterns (Figs 2 and 3). It was important to note that the abilities of drug accumulation were higher in TC than in SMX, which is estimated to be 160 μg g^-1^ (fresh weight) for TC and 18 μg g^-1^ for SMX in *B*. *rapa chinensis*; and 77 μg g^-1^ for TC and 38 μg g^-1^ for SMX in *I*. *aquatica*. TC accumulation in the root of *B*. *rapa chinensis* was significantly higher than the other parts throughout the experimental period, while the root concentration of SMX showed significant difference only after 12–24 hr absorption. In *I*. *aquatica*, although the highest concentration of both antibiotics were also observed in the roots, in several time points the differences were not statistically significant compared to the other parts, possibly due to higher variation of drug concentrations in *I*. *aquatica* roots. Nevertheless, generally both drugs exhibited higher levels in the roots (3–28 fold) compared to leaves and petioles/stems, the only exception was SMX in *I*. *aquatica* in which leave concentrations matched the root concentrations.

Bioaccumulation factors (BAF) were calculated to have a quantitative measure of drug accumulation within different components of the plants (Table 1). The BAFs for TC in *B*. *rapa chinensis* in 24 hrs were 21.1, 1.2 and 0.6 in the roots, leaves and petioles, respectively. In contrast, BAFs for SMX were significantly lower at 0.4 (roots) and less than 0.1 (leaves and petioles). The highest BAF value of *I*. *aquatica* (0.6 in the roots TC) was lower than 1. Based upon the definition of BAF, there is no accumulation of SMX in these plant components.

**Table 1:** The bioaccumulation factor (BAF) of tetracycline and sulfamethoxazole in different parts of *B*. *rapa chinensis* and *I*. *aquatica* at different time points^a^.

Time (h)	*B*. *rapa chinensis*						*I*. *aquatica*					
	TC			SMX			TC			SMX		
	Ro	Pt	Lf	Ro	Pt	Lf	Ro	St	Lf	Ro	St	Lf
0.5	1.6	0.3	0.2	<0.1	<0.1	<0.1	0.1	<0.1	<0.1	<0.1	<0.1	<0.1
3	4.5	0.4	0.5	<0.1	<0.1	<0.1	0.1	<0.1	<0.1	<0.1	<0.1	<0.1
6	14.1	0.4	0.4	<0.1	<0.1	<0.1	0.2	<0.1	<0.1	<0.1	<0.1	<0.1
12	14.8	0.6	1.1	<0.1	<0.1	<0.1	0.4	<0.1	0.1	<0.1	<0.1	<0.1
24	21.1	0.6	1.2	0.4	<0.1	<0.1	0.6	<0.1	0.2	<0.1	<0.1	<0.1

^*a*^Ro, roots; Pt, petioles; Lf, leaves, St, stems

### Metabolism of TC and SMX

A separate study was carried out to ascertain whether any drug metabolites were detectable within plant tissues following a 5-day incubation in the presence of a high concentration (100 μg mL^-1^) of each antibiotic. The analysis revealed no detectable quantities of TC- or SMX-related metabolites in any part of both plants under the described incubation conditions.

### Elimination of TC and SMX

**Fig 4:**
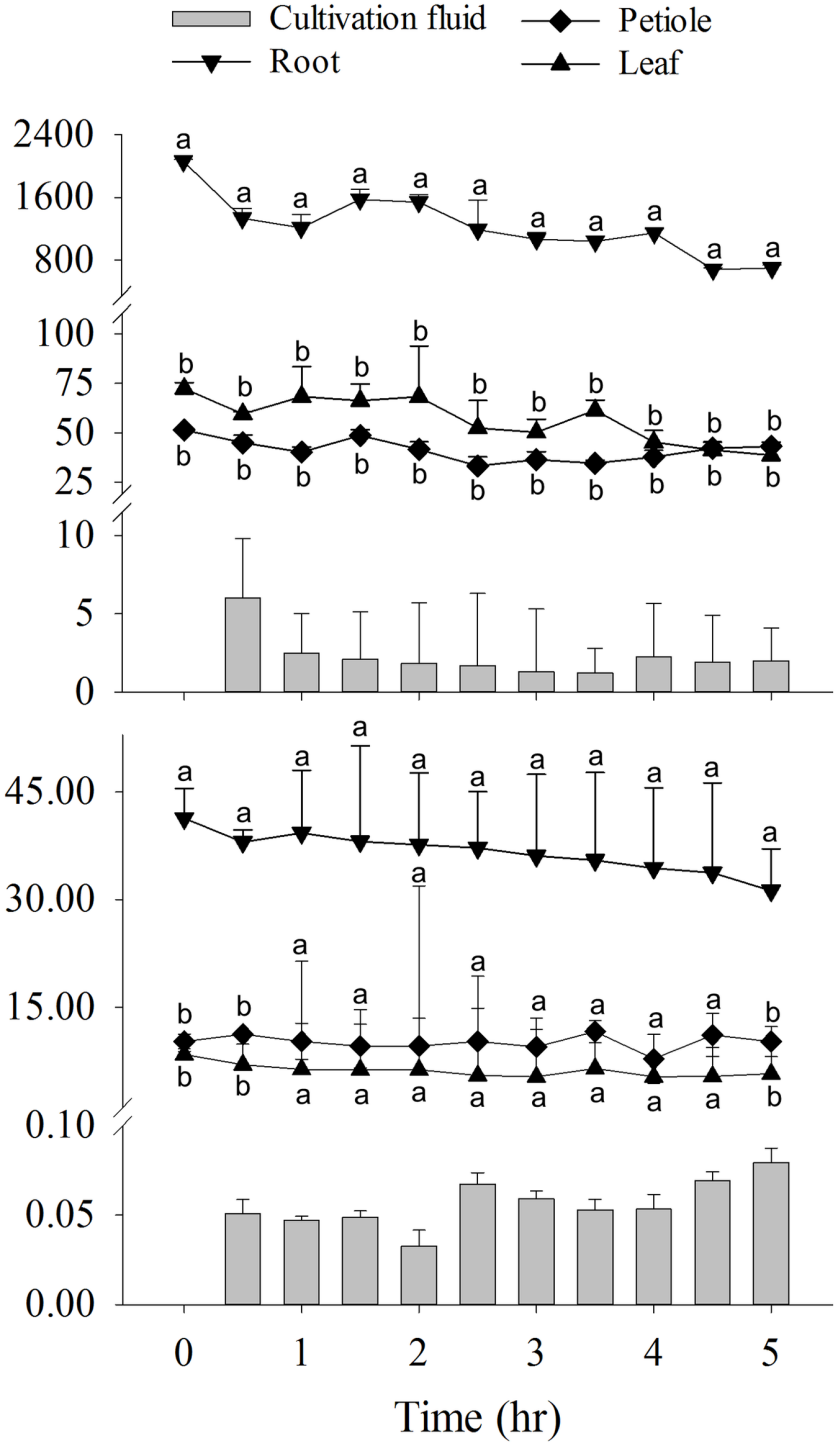
Elimination of TC (top panel) and SMX (bottom panel) by *B*. *rapa chinensis* after 24-hrs of drug absorption. The cultivation fluid was refreshed every 30 min. Values represent means ± SEM. Means in the same time point with different superscripts are significantly different from each other (p<0.05).

Following transfer of the whole vegetable plants into drug-free cultivation fluid, *B*. *rapa chinensis* released back into the fluid 11% (TC) and 8% (SMX) of the drugs that had been absorbed (Fig 2). In comparison, *I*. *aquatica* released 25% (TC) and 28% (SMX) of the total drugs absorbed during the same period (Fig 3). Depletion of stored drug was greatest from the roots and during the initial 30-min period. More specifically, reductions of approximately 25% (TC) and 10% (SMX) for both vegetables were observed from the roots. When cultivation fluid of *B*. *rapa chinensis* was refreshed repetitively, 3% of accumulated TC and 1% of accumulated SMX were released from the plant during each 30-min interval (Fig 4).

### Pharmacokinetic analysis of TC and SMX

Results of non-compartmental pharmacokinetic analysis of TC and SMX presented in Table 2 revealed distinct dissimilarities of these antibiotics in the 2 vegetables. Generally, C_max_ and AUC of TC were larger than those of SMX whereas TC Vss were smaller than SMX in both plant species. Nonetheless, t_½_ and MRT of TC were shorter than SMX in *B*. *rapa chinensis* but were longer in *I*. *aquatica*. CL of both antibiotics were higher in *I*. *aquatica* than in *B*. *rapa chinensis* and higher for SMX than for TC.

**Table 2:** Pharmacokinetic parameters of tetracycline and sulfamethoxazole in the roots of *B*. *rapa chinensis* and *I*. *aquatica* determined by non-compartmental analysis^a^.

Pharmacokinetic parameters	*B*. *rapa chinensis*		*I*. *aquatica*	
	TC	SMX	TC	SMX
C_max_ (μg mL^-1^)	1124	33	35.3	6.2
λ_z_ (hr^-1^)	0.0320	0.0079	0.0134	0.0171
t_½_ (hr)	22	88	52	41
AUC_0-t_ (hr∙μg mL^-1^)	29614	884	935	147
AUC_0-∞_ (hr∙μg mL^-1^)	49280	4591	2973	425
AUMC_0-∞_ (hr^2^∙μg mL^-1^)	1880696	622935	244058	29467
MRT_0-∞_ (hr)	26	124	70	57
CL (mL hr^-1^)	0.089	0.109	0.343	1.27
Vss (mL)	2.3	13.5	24.0	72.9

^*a*^C_max_, maximum drug concentration; λ_z_, terminal rate constant; t_½_, elimination half-life; AUC_0-t_, area under the concentration-time curve from time zero to time t; AUC_0-∞_, area under the concentration-time curve extrapolated to infinity; AUMC_0-∞_, area under the first moment of the concentration-time curve extrapolated to infinity; MRT, mean residence time from time zero to infinity; CL, apparent total body clearance; Vss, volume of distribution at steady state

### Market and field investigation of TC and SMX

Field investigation revealed that higher positive rates (31%) and higher absolute amount (up to 104 ng g^-1^) of TC was recorded in *B*. *rapa chinensis*, while only 2.9% occurrence rate and no more than 10 ng g^-1^ SMX was found (Table 3). In contrast, only 5% of *I*. *aquatica* was found to contain TC with highest concentration of 16 ng g^-1^ and none of SMX was detected.

**Table 3:** The occurrence of tetracycline and sulfamethoxazole in the *B*. *rapa chinensis* (n = 35) and *I*. *aquatica* (n = 37) from local markets and pig farms around Taiwan and their highest concentration detected^a^.

Drugs	*B*. *rapa chinensis*					*I*. *aquatica*				
	Occurrence			Highest conc. (ng g^-1^)		Occurrence			Highest conc. (ng g^-1^)	
	LM	PF	Total	LM	PF	LM	PF	Total	LM	PF
**TC**	30%	40%	31%	104	88	0%	29%	5%	NA	16
**SMX**	3.3%	0%	2.9%	10	NA	0%	0%	0%	NA	NA

^*a*^LM, local markets; PF, pig farms; NA, not applicable.

## Discussion

Widespread use of antibiotics in animal husbandry has been the subject of increasing controversy owing to the potential for negative environmental impacts, including heightened bacterial resistance, disturbances in normal microbial function in soil or water, potential toxicity to plants and wildlife, and risk of contaminating food chains [6,8,10]. Since antibiotics can be accumulated by plants to different extents, they pose potential health risks to consumers who ingest the drug-contaminated vegetables. However, information about the ability of common edible vegetables to absorb antibiotics, their accumulation patterns, and the potential for release back into environment are limited. Although some studies have investigated the absorption and deposition of different antibiotics in plants, including TC and SMX [13,26–31,34–37], no studies have taken from a pharmacokinetics view point, in which absorption, distribution, metabolism, and elimination processes could be determined and modeled for risk assessment. This may largely due to the inability to quantitate a drug releasing process from the plant. The present study was designed to characterize the fate of 2 commonly used veterinary antibiotics, namely TC and SMX, in popular edible vegetables using a pharmacokinetic approach that is used traditionally in animal species. Although plants exhibit numerous structural and functional differences from animals, most plants have enzymes that can metabolize xenobiotics as well as some capacity to eliminate foreign compounds. The present investigation provides a new approach to characterize and quantify the movements of drugs within a plant and between the plants and the environment using traditional pharmacokinetic processes.

Our results demonstrated that drug behaviors in the plants could potentially be explained by pharmacokinetic concept adapted from animal model. Despite the fact that plants contain no blood vessels (central compartment), they do possess a functional comparable organ in the root system that is responsible for uptaking drug molecules for distribution to petioles/stems and leaves. In that sense conceptually the roots could be considered as a central compartment especially when it was proven capable of back-releasing drugs to the environment (Figs 2–4). Non-compartmental model seems to be the reasonable choice for this exploratory phyto-pharmacokinetics analysis since it does not assume any compartment numbers; in addition, because roots steadily and continuously absorbed drugs from the cultivation fluid, intravenous (IV) infusion mode was chosen accordingly as the best available mode.

A significant observation from this study is the remarkable dissimilarity between the pharmacokinetic behaviors of TC and SMX, most notably in the absorption process. TC is uptaken rapidly by hydroponically-grown *B*. *rapa chinensis*, with an average accumulation of 160 μg g^-1^ in the 24-hr period, primarily in in the roots with levels approximately 11-fold greater than those in the incubation fluid. By comparison, only 18 μg g^-1^ of SMX was absorbed into each plant, although also mostly in the roots but there appears to be no net drug accumulation (Table 1, BAF<1). In contrast, although TC also reached at a higher level than SMX in *I*. *aquatica*, its peak concentration was only one-third of the fluid concentration and was 20 times lower than that in the roots of *B*. *rapa chinensis*. Given the much larger capability of both plants to uptake TC than SMX, it was conceivable that the C_max_ and AUC are also larger in *B*. *rapa chinensis*. Consistent with this finding, other studies that investigated the absorption of TCs and SAs under hydroponic conditions also came to the same conclusion. For example, Grote et al. [34] found that chlortetracycline (CTC) was better absorbed than sulfadiazine (SDZ) in wheat. Liu et al. [37] also reported that a common reed was able to absorb OTC to a greater degree compared with SMT.

Differential distributions by drugs and plants were clearly demonstrated by the apparent volume of distribution. The Vss of TC was smaller than those of SMX (Table 2) in both plant species, implying that TC was relatively more restricted to a compartment (the root) than distributed to the other parts of the plant, which is distinct from the case of SMX. These numbers matched well with the data presented in Figs 2 and 3 where smaller difference among SMX concentrations in the roots and the other tissue parts were recorded.

As a primary uptake organ, roots can accumulate these drugs to a greater extent compared to stems/petioles or leaves. The solubility (about 1 g L^-1^) and log K_ow_ (<1) values of TCs and SAs indicates that these drugs are hydrophilic and would be easily accumulated in the roots by passive uptake through apoplast pathway [14]. However, passive diffusion alone can not explain the much higher concentration in the root than in the environment, as were consistently seen in many studies regardless of culture media types (soil, water, or solid agar) [26–30,34–37]. A few factors were known to affect the effective transport of antibiotics from the root to other parts of the plant. The toxicity of xenobiotics to root by reducing water uptake and transpiration [29] or inducing electrolyte leakage from the root [24] were proposed. Nevertheless, broader drug distributions [13,28,37] was also reported which implies that the distribution pattern may depend on multiple factors including plant species, physicochemical properties of the drugs, and other external factors. The current study suggested not only drug-specific but also plant-specific tissue distribution (Figs 2 and 3) with strong indication that petioles/stems may act primarily as a conducting channel [37] rather than storage site.

Once accumulated in plant tissues, most organic contaminants appear to undergo some degree of biotransformation before undergoing sequestration in vacuoles or becoming bound to insoluble cellular structures as part of detoxification process [14,41]. Epimers of TC (4-epiTC) and CTC (4-epi-CTC) have been detected in the leaves of TC- and CTC-treated pinto bean, respectively, after exposure to the antibiotics for as short as 24 hr [42]. In contrast, 4-hydroxy-SDZ was found in the willow (roots, stems, and leaves), maize (roots and stems), and common hazel (roots) treated with SDZ. Only N-acetyl-SDZ was detected at trace levels (≤0.02 mg kg^-1^) in willow leaves after exposure to the antibiotics for 40 days (willow and maize) or 2 months (common hazel) [24,29]. The present study could not identify any drug-related metabolite within a 5-day cultivation period, which may indicate that under current conditions TC and SMX are not subject to significant biotransformation.

The elimination mechanism of xenobiotics in plants is anatomically different from those of animals. Unlike most animal species, vegetables have no specific organs for drug excretion. Instead, plants translocate foreign compounds into vacuoles and cell walls through sequestration or compartmentalization [14,41]. The elimination of antibiotics from plant tissues into the surrounding environment has remained a relatively unstudied area, passive release in relatively hypotonic fluid is a possibility. Our results provide evidence that TC and SMX may be released either actively or passively from the plants into the surrounding solution. Differential pharmacokinetics was also observed in the elimination process. In *B*. *rapa chinensis*, t_½_ and MRT of TC were shorter than those for SMX, suggesting that TC was more readily eliminated; in contrast, both parameters appeared longer for TC in *I*. *aquatica*. Overall, the ability of plants to eliminate TC and SMX was evident, but appears to have limited impact on drug reduction since their elimination t_½_ from the roots are relatively long (in the range of 22–88 hr). Drug elimination from the leaves and petioles/stems seemed to play an insignificant role but reduction in concentrations were detectable. The elimination was more evident when cultivation fluid was refreshed every 30 min (Fig 4), at which time similar amount of drugs (about 3% and 1% of the absorbed TC and SMX, respectively) were constantly released back into the blank fluid, suggesting that drug elimination take place through a regulated process via a mechanism that has not been identified. One possible explanation lies in the drug-protein interactions such that only the free fraction of drug is available for release (into the surrounding water) until certain equilibrium is established.

Distinguishable pharmacokinetic characteristics are noted when comparing the current results to those in human body, for instance, elimination t_½_ of both antibiotics in the vegetables are much longer (22–88 hrs versus 8–11 hrs in human [43]); and the Vss is higher in SMX than in TC in plants (Table 2) while the reverse was true for human (0.1–0.2 L kg^-1^ for SMX [44] and 1.3 L kg^-1^ for TC [45]). While these differences can be conceivably attributed to the inherited anatomical differences between animal and plant kingdom, our study highlighted the possibility to assess drug behaviors within a plant by applying pharmacokinetic parameters such as C_max_, AUC, Vss, t_½_ and MRT and underscored the importance for more studies. Much are needed in the understanding of basic physiological properties, metabolic processes and elimination pathways characteristic for the plants. Problems associated with the direct application of the classical pharmacokinetic model for plants, in this case non-compartment analysis, were discussed below. In principle, the concentration profile in the post-exposure period (24–36 hr) is not a mono-exponential but bi-phasic decline (Figs 2 and 3). This is likely due to plants do not have a kidney-equivalent organ for active drug elimination; the elimination will be mainly attributed to dissipation from roots to the medium and catabolic decay in tissue. As such, non-mono exponential decline at the terminal phase could potentially render a misdirected estimation of Lambda-z and AUC depending on the model. Similarly, fraction of extrapolated area (ratio of AUC_t-∞_ to AUC_0-∞_) might be increased (Table 2). Alternative model such as multi-exponential model could be considered based on the elimination profile. Nevertheless, the number generated by non-compartmental analysis matched the general interpretation of the results and conceptually demonstrated the feasibility of conducting phyto-pharmacokinetic studies.

In parallel with our controlled experimental studies, we have conducted a preliminary field investigation on residue levels of these drugs in studied vegetables. The highest concentration of TC detected was 104 ng g^-1^ whereas no more than 10 ng g^-1^ SMX was observed. In combination, the present study showed the presence of these drugs at over a hundred ppb level and can be accumulated to a ppm level in the edible parts within a short period (hrs) of exposure, as seen with TC as parent compound in the leaves. Therefore, potential threat to consumers remain, especially when the drug-contaminated sewer water is used for irrigation or when water flooded the contaminated soil of vegetable field.

## Conclusions

To the best of our knowledge, this is the first phyto-pharmacokinetic study designed to investigate pharmacokinetic behaviors of antibiotics (TC and SMX) in short-living vegetables (*B*. *rapa chinensis* and *I*. *aquatica*) grown under hydroponic condition. We demonstrated for the first time that pharmacokinetics approaches commonly used in animal model could be used to at least partially describe drug behaviors in the plants from its absorption to elimination. Differential pharmacokinetic characteristics between 2 antibiotics in the same plant and for the same drug in different plants were discovered. It is still pre-mature for our results to perform a complete pharmacokinetic modeling. Shortcomings and potential hindrance are proposed for future optimization of pharmacokinetic techniques in plant studies. Our results clearly demonstrated the practicability of pharmacokinetics analysis as a promising tool for future better understanding of fate of chemicals in agronomic fields and potentially in grasslands and forest. Further studies to delineate the underlining factors affecting the pharmacokinetics of different drug classes in the vastly varied plant species are warranted.
